# Characterizing Nature and Participant Experience in Studies of Nature Exposure for Positive Mental Health: An Integrative Review

**DOI:** 10.3389/fpsyg.2018.02617

**Published:** 2019-01-04

**Authors:** Michael R. Barnes, Marie L. Donahue, Bonnie L. Keeler, Cameron M. Shorb, Tara Z. Mohtadi, Lacy J. Shelby

**Affiliations:** ^1^Department of Forest Resources, University of Minnesota Twin Cities, Saint Paul, MN, United States; ^2^Institute on the Environment, University of Minnesota Twin Cities, Saint Paul, MN, United States; ^3^Humphrey School of Public Affairs, University of Minnesota Twin Cities, Minneapolis, MN, United States; ^4^The Good Food Institute, Washington, DC, United States; ^5^Earth Institute, Columbia University, New York, NY, United States; ^6^New York City Department of Transportation, New York City, NY, United States

**Keywords:** environmental psychology, nature-exposure, mental health, urban design, public health

## Abstract

A growing number of studies demonstrate significant associations between nature experiences and positive mental health outcomes (e.g., improved mood, decreased stress). However, implementation of this research by practitioners in fields such as urban design or public health has been limited. One reason for this is that it remains unclear what elements of nature and types of participant experience are consistently associated with mental health benefits. As a result, decision-makers who aim to enhance mental health in cities have little guidance about which elements of nature and types of experiences in natural areas may lead to positive mental health outcomes. We reviewed 30 studies with 41 distinct exposures in nature that elicited positive mental health benefits and characterized the elements of nature found at these sites, as well as aspects of participants’ experience. Elements of natural areas considered include: forest, managed grass, and water as dominant land cover types, specific water features (e.g., small ponds, fountains) and built features (e.g., trails, paths). The majority of the studies we reviewed assessed the experiences of individuals (vs. in groups) participating in walks during warmer seasons. Most studies did not describe the “nature of the nature” associated with positive mental health outcomes. We contacted authors and used Google Earth imagery to reconstruct the specific natural elements, landscape typology, and site adjacencies present in past studies. We recommend specific ways researchers could better and more transparently document important elements of nature and participant experience in study design and reporting that will enhance the planning and design relevance of their work.

## Introduction

Urbanization has been associated with increased rates of mental illness in cities worldwide (Okkels et al., 2017). In response, there is a growing interest and urgency in understanding how the urban environment impacts human health and well-being (Hartig et al., 2014; Shanahan et al., 2015; Frumkin et al., 2017). Broadly, natural spaces have been associated with a wide range of health benefits, such links have been consistent and generally well-understood (van den Bosch and Sang, 2017). Evidence from environmental psychology using a variety of methodologies (including self-report, psychophysiological assessments, and others), have demonstrated that contact with nature enhances positive affect, self-esteem, and cognitive functioning (Barton and Pretty, 2010; Zelenski and Nisbet, 2014; Bratman et al., 2015a; among others). These effects have also been demonstrated to occur across a wide range of demographic groups and sub-populations (Faber Taylor and Kuo, 2011; Ward Thompson et al., 2012; Beyer et al., 2014; Wu et al., 2015). Despite this no studies have yet to connect the nature of the nature to mental health outcomes. As such, specific findings from research on the mental health benefits of nature have yet to be implemented by practitioners such as landscape architects, urban planners, and public health officials, in part because it remains unclear what elements of nature exposure or types of nature experiences and landscapes provide mental health benefits (Gomez-Baggethun and Barton, 2013).

Interdisciplinary research and applied work often requires insights or integration that require alternative approaches and new types of data collection. Our work is unique in that we take an applied perspective on past work on the mental health benefits of urban nature that reveal key shortcomings that are needed to translate research to actionable design solutions for designers and planners. Charged with implementing nature-based solutions and enhanced nature-based design features in the urban context, designers and planners rely on evidence-based research to advance programmatic and policy goals for cities. Outcomes for achieving improved mental health are accessible to the designer and planner, but only when research clearly identifies the components, qualities, and features of landscapes and cityscapes experienced by participant’s. Adoption of research in this area by the design profession requires enhanced descriptions of the qualitative features, environmental conditions, and quality of the nature as experienced by participants. Terminology used by designers can easily be incorporated when describing the experience of participants in studies in nature. Examples of where such design language could be incorporated include: enhanced definitions of landcover types, quantifying the density of vegetative cover, describing proximity to other features like rock outcroppings, wildlife habitat, built structures, and offering dimensions of trails and paths and their surface types. Our aim was to evaluate how much of the current body of mental health research on urban nature could be put into practice in the design of streetscapes, urban parks, or other public spaces. This requires knowing something about the “nature of the nature” that was previously found to have positive associations with mental health benefits.

## Methods

### Literature Review

We sought to identify and characterize the elements of natural environments and participants’ experiences in them by reviewing relevant nature-exposure studies that demonstrate a positive mental health benefit. We conducted an integrative review of the literature to assess common elements, locations, and features used in nature-exposure research. We compiled a database of nature-exposure studies that demonstrated positive mental health outcomes to assess what types of green spaces have demonstrated these benefits.

We systematically reviewed reference lists from all relevant meta-analyses and review papers that investigated the mental health impacts of urban nature exposure and were published by 2016 (these were: Barton and Pretty, 2010; Bowler, 2010; Thompson Coon et al., 2011; Bratman et al., 2012; Keniger et al., 2013; Hartig et al., 2014; Kuo, 2015; McMahan and Estes, 2015; van den Berg et al., 2015). We supplemented the resulting list of studies with nature-exposure research known to our team but not yet included in existing meta-analyses or reviews (these additional studies included: Beil and Hanes, 2013; Bratman et al., 2015a,b; Ochiai et al., 2015; Korpela et al., 2016; Scopelliti et al., 2016; Wilson et al., 2016).

After compiling relevant reference lists and studies, we removed duplicates and included studies that met the following five criteria:

(1) Conducted original, primary research on participants’ response to nature using tests of affect (e.g., mood), cognitive function (e.g., memory) or other validated well-being metrics;
(2) Tested responses to a real-life nature exposure, as opposed to *simulated* nature exposure (e.g., via videos or photographs) or methods that included aggregate measures of nature (e.g., relative greenness);
(3) Found positive results from nature exposure, given our interest in understanding the types of nature exposure that *benefit* mental health;
(4) Provided enough information for us to identify the geographic location of the nature exposure; and
(5) Was published or available in English.

**FIGURE 1:**
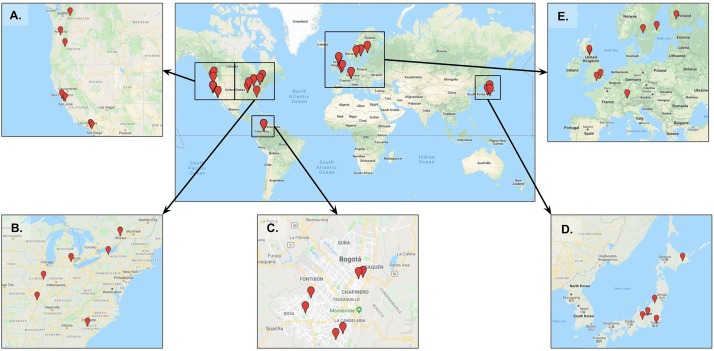
Nature-immersion exposure locations. Locations of 41 distinct nature-exposures, visualized at a global scale and then by region in **(A–E)**, as follows: **(A)** corresponds to the Western United States region; **(B)** to the Eastern United States and Canada; **(C)** to Bogota, Colombia; **(D)** to Japan; and **(E)** to Europe. Explore the full map in more detail at: http://bit.ly/natureexpsitesmap (Basemap data 2018 Google, SK telecom, ZENRIN).

After screening studies for these criteria, we identified 41 unique nature-exposure experience locations worldwide (Figure 1) based on 30 peer-reviewed and published studies (for full reference list of studies included, see Supplementary Table S1). We synthesized information, after abstraction, about the methods and nature-exposure sites in each paper for our analysis.

### Indicator Selection

Insights from an interdisciplinary team with expertise in environmental psychology, ecosystem services, ecology, human dimensions of natural resource management, urban planning, and landscape architecture helped generate a list of potentially relevant indicators that could characterize the diversity of general study elements, study sites and participant experiences in nature-exposure studies (Table 1 includes a full list of the variables characterized). The selected indicators spanned four broad categories:

**Table 1:** Summary indicators of nature-immersion exposure.

Summary indicators: *Study and exposure characteristics*	Results^∗^	Summary indicators: *nature characteristics*	Results^∗^
**Park or nature area size** *(ha)*	Small (<20 ha): mean = 6 ha; *n* = 12 or 29.3% Medium (20–100 ha): mean = 44 ha; *n* = 13 or 31.7% Large (100–5,000 ha): mean = 837 ha; *n* = 10 or 24.4% Very Lg. (>5,000 ha): mean = 137,500 ha; *n* = 2 or 5.9% Unspecified: *n* = 4 or 9.8%	**Urban density** *(*i.e*., physical density of buildings within a 1-mile radius)*	High = 27 (65.9%) Medium = 7 (17.1%) Low = 7 (17.1%)
**No. of participants** *(people, count)*	Mean = 44 people (*n* = 41) Minimum = 4 Maximum = 112	**Dominant land cover**	Forest = 20 (48.8%) Managed grass = 15 (36.6%) Grassland = 2 (5.9%) Water = 2 (5.9%) Mixed = 2 (5.9%)
**Duration^∗∗^** *(mean)*	Mean = 63 min (*n* = 29) Minimum= 10 min Maximum = 360 min	**Large built structures** *(*e.g*., picnic pavilion, welcome center)*	Yes = 29 (70.7%) No = 12 (29.3%)
**Social context**	Alone = 19 (46.3%) In a group = 11 (26.8%) Variable = 9 (22.0%) Unspecified = 2 (5.9%)	**Small built structures** *(*e.g*., benches, picnic ‘s, playgrounds)*	Yes = 32 (78%) No = 9 (22.0%)
**Activity**	Walking = 22 (53.7%) Sitting = 4 (9.8%) Variable = 10 (24.4%) Other = 5 (12.2%)	**Trails**	Yes = 37 (90.2%) No = 4 (9.8%)
**Season**	Summer = 13 (31.7%) Spring = 7 (17.1%) Fall = 5 (12.2%) Winter = 2 (5.9%) Multiple = 2 (5.9%) Unspecified = 12 (29.3%)	**Outdoor sports facilities** *(*e.g*., basketball, soccer field, ice rink)*	Yes = 9 (22%) No = 32 (78%)
**Map included**	Yes = 10 (24.4%) No = 30 (73.2%) Provided by request = 1 (2.4%)	**Water features**†	Built = 9 (22%) Natural = 14 (34%) Both built and natural = 3 (7%) No water features = 13 (32%) Unknown = 2 (5%)
**Photo(s) included**	Yes = 10 (24.4%) No = 30 (73.2%) Provided by request = 1 (2.4%)		
**Outcome variable(s)^∗∗∗^**	Affect/mood = 37 (90.2%) Cognitive function = 11 (26.8%) Physiological = 13 (31.7%) Other = 7 (17%)		


*^∗^Presented in frequency of occurrence, unless otherwise noted. ^∗∗^n = 29; exposures with multi-day (outliers), variable, or unspecified duration were not included in the calculation. ^∗∗∗^Percentages are calculated related to the total number of exposures (n = 41), studies can include multiple outcome variables. †Two unknown exposures are due to the exact walking path being unknown and taking place in very large wilderness areas.*

(1) Study design (e.g., number of participants, participant groupings, and response variable);
(2) Participant experience characteristics (e.g., social context, duration, type of activity, seasonality);
(3) Geographic location (e.g., park name, country, region);
(4) Landscape features (e.g., land cover types, trails, water, built park amenities, and built disamenities such as busy roadways).

### Data Collection and Analysis

We collected location information and populated selected exposure site variables first through included information present in each study. In cases where we could not determine exact locations from the information provided in-text, we first contacted authors for clarification, maps of routes that participants took, or additional photographs of the site. Then additional detailed supplementary information about site adjacencies and exposures were identified and analyzed using tools such as: satellite imagery, spatial measurement tools, Street View or panoramic eye-level imagery along roads and some pedestrian paths, and user-submitted geotagged photographs from Google Maps, Google Street View, and Flickr.com respectively. Landcover types were assessed using a visual estimate if one landcover type covered more than half of the study area, we recorded it as the dominant landcover. If no single landcover type covered more than half of the study area, the dominant landcover type was listed as “mixed.” Water features were identified if a water element was present, this provides additional specificity compared to water categorized as landcover type. Urban density was assessed using an estimated relative physical density of built structures and dwelling units within a 1-mile radius around the edges of the natural area; analysts visually coded this indicator in categories of low (less than five buildings), medium (between five and 20 buildings), and high (more than 20 buildings) density. We also collected information related to study response variables, methods, and participant populations, these variables were not included in our analysis due to being out of scope for the current work as well as the generally broadly positive effects of nature on mental health across diverse demographic groups, however, this information is included in our Supplementary Table S1.

## Results

Table 1 summarizes study characteristics, type and duration of nature exposure, and physical characteristics of the natural setting used in each experiment. Almost three-quarters (73.2%) of the studies did not include both a map and photos of the nature-exposure locations in addition to lacking a robust description of the exposure site. This means that most studies largely did not report the type, size, scale, diversity, or composition of the nature that may have been experienced, nor the type of constructed amenities within green space (such as trails or benches) experienced by study participants. The following results therefore contain a combination of in-text provided and additional data using the tools described in the previous section. Those exposures where nature-exposure locations could be determined were distributed across natural areas of various sizes, ranging from small parks (6 ha on average) to large parks (837 ha on average), with two outliers of very large wilderness areas (137,500 ha averaged). Specific features of nature itself were relatively consistent with most exposures containing trails (90.2%) as well as both large and small built structures, 70.7 and 78% respectively. The presence of water features was more varied however and split between built (*n* = 9), natural (*n* = 14), or no water features of either type (*n* = 13). Almost one third (29.3%) of the studies did not provide any information regarding the season the exposure took place in. The warmer seasons where individuals are most likely to be outside (summer 31.7%, spring 17.1%) were more frequently used than cooler seasons (fall 12.2%, winter 5.9%). Participants were frequently walking during the exposure (53.7%), with few sitting (9.8%), or doing more vigorous activities such as hiking or biking (12.2%). Notably, response variables that measured affect/mood were the most common. The average duration of a nature exposure was just over an hour (63 min), with a wide range between a minimum duration of 10 min, and a maximum of 360 min. Most often participants experienced nature-exposures alone (46.3%) rather than in a group (27.8%). Full results for each exposure included in the review are available in Supplementary Table S1.

## Discussion

Within many of the studies on the benefits of nature experience, descriptions of the elements of nature associated with mental health benefits are understudied and underreported. We sought to understand how well nature-exposure studies characterized these elements. This integrative-review of nature-exposure studies elucidated three main categories of findings which are discussed in more detail in the following sections.

(1) Identification of the key elements of nature which elicited mental health benefits that individuals may have been exposed to.
(2) Identification of common participant experience elements (individually, walking, summertime, etc.).
(3) Identification of the common broader contextual elements surrounding exposure sites that individuals may have been exposed to.

### Key Elements of Urban Green Space

Our review identified specific elements that were present in the majority of studies that found positive mental health benefits. Almost universally green spaces contained a trail of some kind, either gravel/dirt or paved. This is not unusual given that paths help direct flow, and guide individuals through a space, or to a place within a space and are a common design element (Lynch, 1960). Both small and large built structures were present in most green spaces and afforded some type of amenities to the natural spaces. It should be noted however that it’s unclear in the descriptions of participants experiences within studies whether any of the participants used such amenities, which should be included in future work. Valuable to designers and planners would be to understand whether additional amenities or features can support or enhance an individual’s willingness to extend the duration of their exposure to nature, and whether or not it enhances or detracts from the experience. Most green spaces did not contain a formal sports area within them.

From existing evidence it’s unclear whether such formalized, generally single-use forms of urban green space elicit similar benefits as other forms of nature as they potentially don’t share many of the elements found in the current review that are associated with such benefits (Francis et al., 2012). Another common aspect that was common to the majority of green spaces was the presence of water features either built or natural. This would be consistent with previous work whereby water features promoted greater well-being (Völker and Kistemann, 2011).

Finally, green spaces that elicited mental health benefits could be found across a gradient in terms of sizes ranging from a small 1 ha city park to a 159,000 ha wilderness area. As urbanization intensifies globally, the impact of smaller pocket parks and even streetscaping in the form of planters and street trees could be critical elements for improving mental health for urban residents. A small amount of recent studies investigating street trees and pocket parks have found positive associations in terms of health and well-being (Nordh and Østby, 2013; Kardan et al., 2015; Taylor et al., 2015), but these smaller forms of urban nature that individuals have daily contact with are relatively understudied.

### Participant Experience Elements

Most often studies exposed participants alone, especially when those studies were experimental. However, cross-sectional studies, in contrast, were more diverse and included participants being on their own and in groups, often due to approaching individuals who were already using the green space. In addition to social context, the type of activity was consistent across studies, with walking being the most common. Again, it could be hypothesized that higher levels of inclusion of participant activity information in studies was methodologically driven. Given that reporting such information is common practice in psychological studies as part of a robust methodology. An aspect of participant activity which is not reported is additional context related to describing the actual *experience* of individuals. For example, studies reported that participants walked alone, but did not provide additional context around who or what they might have encountered and interacted with, and where their attention was focused during the exposure. Duration of exposure was also an interesting aspect, with a range from 10 min to over 360 min in a single dose. The finding of an ideal ‘dose’ has been a topic of discussion previously (see Bratman et al., 2012). Related to duration is also frequency of contact with nature, which to date research has found mixed results as to whether or not frequency provides additive well-being benefits or not (Korpela and Yién, 2007; Lafortezza et al., 2009). Seasonality was the last common element of participant experiences, and was notably an element which was reported on a fairly consistent basis in the reviewed studies. We found a noticeable bias toward spring and summer seasons in reviewed studies. Most locations where nature-exposures took place were in temperate regions (Figure 1) that have a wide range of seasonal variability. With most studies reporting seasonality, it is one of the areas in which further work can be done right away posing the question whether mental health benefits of nature persist in winter when significant changes in the natural landscape occur. Most studies included the four elements of participant experiences (social context, dose, activity, seasonality), however, clarity in the social context, and the specific types of interactions that happened to or among participants during their nature-exposure were largely unreported and should be improved in future work. This can also be seen in the dimension of seasonality as perhaps certain types of interactions are more common in different seasons, and thus could alter the experience of nature.

### Broader Contextual Elements of Urban Green Spaces

First, the broader context in which nature experiences take place are commonly not reported in nature-exposure studies. The issue of addressing the broader context in which such nature exposures take place starts with the lack of specificity in defining the boundaries of the green spaces themselves. Few current studies under review reported the boundaries or definitive size of the green spaces. Green space size was most often reported for spaces that had defined boundaries (e.g., a contained urban park) compared to those with more amorphous borders (e.g., rural natural area). The lack of defining boundaries and size makes it difficult for designers and planners alike to assess the potential for proximate sources of nuisances such as noise or pollution. Descriptions and photos illustrating proximity to structures and built form, land use type and transportation infrastructure were key missing features that are essential in supporting actionable solutions for designers, especially for natural spaces located in highly dense urban landscapes. Building density proximate to green space may reduce the positive outcomes achieved in green spaces designed for positive mental health benefits due to increased nuisances. The nearby density and other sources of auditory and olfactory nuisances (e.g., trains, factories) could impede the effectiveness of urban nature to provide mental health benefits for residents (Lyytimäki et al., 2008; Tzivian et al., 2015; Hammersen et al., 2016). Another issue affecting the broader context of urban green spaces is the relative density of public green spaces that occur within a city. Specifically a question that arises related to this issue of public green space density would be if the effects of green spaces on mental health are intensified in low green space density cities vs. greater green space density. Describing and ideally inventorying the broader context in which nature exposures occur would be helpful in identifying potential sources of stressors, nuisances, and density issues that could play a role in driving the observed effects of nature on mental health benefits while offering the designer and planner key design direction when developing and planning nature space.

Understanding both specific elements and the broader contextual aspects related to public urban green spaces are not only important for those individuals who actually experience such spaces first hand, but also for those who experience such spaces through viewing them. Recent work has connected views of nature to mental health and well-being (Kaplan, 2001; Pretty, 2004; Honold et al., 2016). Therefore a deeper understanding of the specific elements and broader contextual aspects of urban green spaces can have a much greater impact beyond those individuals directly exposed to the space itself.

### Recommendations

In an effort to understand specific features that elicit mental health benefits, provide practitioners with easily accessible and readable information, and increase transparency in nature-exposure research, we provide the following list of actionable recommendations that could be adopted by those involved in future study design and reporting of nature-exposure for mental health research.

#### Participant Experience

• Ensure exposure experience descriptions are specific including:
   ◦ Duration of nature-exposure experience for each participant
   ◦ Information on whether participants were alone or with others
   ◦ Specific activities of participants (e.g., walking slowly and surveying nearby vegetation)
   ◦ Map and provide a specific description of exposure route (if mobile) or exact location (if stationary).

#### Exposure Location and Geography

• Identify and report nature-exposure exposure sites by most commonly known name (e.g., Golden Gate Park), or location in relation to another landmark (e.g., campus green space west of Coffey Hall, University of Minnesota), if no formal name exists.
• Include location and map of where exposure took place and a description of the surrounding area. This may include sights, sounds, and smells.
• Include proximity, porosity/imperviousness, and relative density of adjacent structures.

#### Environmental Context and Natural Elements

• Photograph surroundings that participants would view or encounter during exposure.
• Describe nearby built and natural features that participants may experience.
• Include not only amenities but also stressors, such as:
   ◦ Sources of noise (e.g., nearby railroad lines, airports, highways, etc.)
   ◦ Sources of strong odors (e.g., factories, construction, restaurants, etc.)
   ◦ Other unique factors or stimuli that may influence participant experience

#### Overarching Recommendations

• Use accessible tools including GIS software and Google Maps to summarize natural and neighborhood metrics of exposure sites
• Explore opportunities for conducting exposure studies in locations where existing evidence is lacking, particularly in the Global South
• Encourage a broader range of seasonal experiences and exposure in nature as well as time of day and duration.

Our findings and these recommendations can be taken as a call to continue improving how we understand what factors are associated with mental health benefits of nature and what causal mechanisms may be responsible. The recommendations provide a starting point for understanding the complex relationship between nature and well-being. Limitations in our own work given gaps in available, in-text descriptions that made it difficult to interpret or code specific elements present in the mental health and well-being benefits based on in-text study descriptions alone. Because of these gaps, we relied on coding a large number of locations and landscape elements ourselves using available online resources, including Google Maps or Street View.

## Conclusion

We provided an assessment of the current state of knowledge of nature-exposure studies that resulted in reported positive mental health benefits. Overall, we found that studies of nature exposure for mental health generally described participant experiences better and more comprehensively than information on either location or landscape context. A significant first step then is providing greater detail in studies as to the nature of the nature in order to assess features and elements that can measurably be attributed to enhancing an individual’s sense of well-being. Additionally, such details will assist to enhance the design practice, encourage interdisciplinary research, and ultimately design better public spaces.

## Author Contributions

MB and MD wrote the manuscript. BK conceived of and supervised the project and manuscript through its creation. CS and TM gathered and analyzed data. LS provided expert advice and guidance for practitioners. All authors provided substantive feedback throughout.

## Conflict of Interest Statement

The authors declare that the research was conducted in the absence of any commercial or financial relationships that could be construed as a potential conflict of interest.
